# Comparability of a Three-Dimensional Structure in Biopharmaceuticals Using Spectroscopic Methods

**DOI:** 10.1155/2014/950598

**Published:** 2014-05-22

**Authors:** Víctor Pérez Medina Martínez, Mario E. Abad-Javier, Alexis J. Romero-Díaz, Francisco Villaseñor-Ortega, Néstor O. Pérez, Luis F. Flores-Ortiz, Emilio Medina-Rivero

**Affiliations:** ^1^Unidad de Investigación y Desarrollo, Probiomed S.A. de C.V. Cruce de Carreteras Acatzingo-Zumpahuacán S/N, Tenancingo, 52400 Mexico, MEX, Mexico; ^2^Departamento de Ingeniería Bioquímica, Instituto Tecnológico de Celaya, Avenida Tecnológico y Antonio García Cubas S/N, 38010 Celaya, GTO, Mexico

## Abstract

Protein structure depends on weak interactions and covalent bonds, like disulfide bridges, established according to the environmental conditions. Here, we present the validation of two spectroscopic methodologies for the measurement of free and unoxidized thiols, as an attribute of structural integrity, using 5,5′-dithionitrobenzoic acid (DTNB) and DyLight Maleimide (DLM) as derivatizing agents. These methods were used to compare Rituximab and Etanercept products from different manufacturers. Physicochemical comparability was demonstrated for Rituximab products as DTNB showed no statistical differences under native, denaturing, and denaturing-reducing conditions, with Student's *t*-test *P* values of 0.6233, 0.4022, and 0.1475, respectively. While for Etanercept products no statistical differences were observed under native (*P* = 0.0758) and denaturing conditions (*P* = 0.2450), denaturing-reducing conditions revealed cysteine contents of 98% and 101%, towards the theoretical value of 58, for the evaluated products from different Etanercept manufacturers. DLM supported equality between Rituximab products under native (*P* = 0.7499) and denaturing conditions (*P* = 0.8027), but showed statistical differences among Etanercept products under native conditions (*P* < 0.001). DLM suggested that Infinitam has fewer exposed thiols than Enbrel, although DTNB method, circular dichroism (CD), fluorescence (TCSPC), and activity (TNF**α** neutralization) showed no differences. Overall, this data revealed the capabilities and drawbacks of each thiol quantification technique and their correlation with protein structure.

## 1. Introduction

Rituximab and Etanercept are biopharmaceutical recombinant proteins used for the treatment of non-Hodgkin lymphoma and rheumatoid arthritis [1, 2]; and as a monoclonal antibody and a fusion protein they are composed of a pair of heavy and light chains and a homodimer with several cysteines, respectively. Both proteins are assembled with inter- and intrachain disulfide bonds that are established during their biosynthesis in the endoplasmic reticulum of recombinant CHO cells [3–5]. Once folded, glycosylated within the cell, and secreted into culture medium, recombinant proteins can be subjected to physicochemical stress, either during their residence in the cell culture media or during the steps of purification, formulation, filling, and storage, which stem mainly from changes in pH, temperature, and ionic strength [6, 7]. The degree of modification determines the stability of the protein, which may expose buried cysteine residues or broken disulfide bridges, allowing for the measurement of the free thiols as a quality attribute for ensuring the safety and efficacy of a biopharmaceutical product. During the last years spectroscopic techniques used for recombinant protein characterization are gaining interest in order to identify specific structural properties, folding phenomena, and stability based on the absorptivity response, fluorescence, and light dispersion effects [8–10].

Several protocols for quantifying free thiols have been described in the past to study protein structure and stability. That is the case of Ellman's technique, first described in 1959 [11]. However, to the best of our knowledge, there are no validation protocols and criteria for the measurement of free thiols in biopharmaceuticals, and there is no correlation between the structural conditions and biological activity of the biotherapeutic protein and their exposure of thiol groups.

Colorimetric methods, like 5,5′-dithionitrobenzoic acid (DTNB), are useful to analyze free thiols above the nanomolar range but at the expense of large protein quantities [12, 13]. To overcome this, DyLight Maleimide (DLM) can be used for the nanomolar range [14], taking advantage of their fluorescence properties and selectivity against thiol groups, well known for Maleimides [15, 16]. Both orthogonal techniques can provide a wider concentration range for thiol analysis and a verification of the total content or trend observed for a protein, when analyzed under different conditions.

Native, denaturing, and denaturing-reducing conditions allow for the quantification of the free cysteine's thiols coming from degradation, unfolding, or broken disulfide bridges buried in the protein and the total cysteines content within the protein, respectively. In every case, a correlation against the integrity of the protein structure can be done by the use of spectroscopic techniques such as circular dichroism (CD) [15] and intrinsic fluorescence lifetime measured by time-correlated single photon counting method (TCSPC) [16]. Protein identity and oxidation can be tested from the comparison of the theoretical content of cysteines that comes from the amino acid sequence. This information together contributes to the physicochemical characterization and the establishment of quality attributes and comparability criteria among products.

The use of suitable methodologies for intended purpose, mentioned before, plays an essential role in biopharmaceutical companies, as they are feasible tools for bioprocess development, formulations design, and stability studies in order to define product shelf life.

Here we present a physicochemical comparability study using two orthogonal thiol quantification methods, correlated with other spectroscopic and biological methods, and its validation. Troubleshooting is given for both techniques.

## 2. Materials and Methods

### 2.1. Reagents

L-cysteine HCl, Ellman's reagent, DyLight 488 Maleimide, and Slide-A dialysis cassettes were obtained from Thermo Scientific (Cincinnati, OH, USA). Monobasic and dibasic sodium phosphate, sodium citrate, citric acid, mannitol, sucrose, and sodium azide were obtained from J. T. Baker (Center Valley, PA, USA). EDTA, dithiothreitol (DTT), guanidine hydrochloride (GdnHCl), Tris-HCL, polysorbate 80, crystal violet, dimethyl sulfoxide (DMSO), phenylisothiocyanate (PIT), and heat inactivated fetal calf serum were obtained from Sigma-Aldrich (Saint Louis, MO, USA). Human serum complement was obtained from Quindel (San Diego, CA, USA); Somatropine CRS (Batch 3, Code: 50947000, Id: O12YRG) was obtained from EDQM Ph. Eur (Strasbourg, France). Human serum albumin (HSA) batch 4330200029 was purchased from CSL Behring AG (King of Prussia, PA, USA). Glatiramer acetate (GA) batch AGPP12001 was obtained from Probiomed S.A. de C.V. (Mexico City, Mexico) and 10 kD protein Marker part number A26487 for cIEF batch M205297 was obtained from Beckman Coulter (Brea, CA, USA). DMEM medium supplemented with glutamine 6 mM and pyruvate 1 mM was obtained from Gibco, Life Technologies (Waltham, MA, USA). A375 human melanoma and WIL2-S lymphoma cell lines were purchased from ATCC (Manassas, VA, USA) and maintained in a humidified chamber at 37°C with 5% CO_2_.

NAP-5 Gel filtration columns were obtained from GE Healthcare (Buckinghamshire, UK). 30 kDa centricons were obtained from EMD Millipore (Billerica, MA, USA). Neuraminidase was obtained from New England Biolabs (Ipswich, MA, USA). Mabthera batches, B60360, B60420, B60480, B60490, B6074B04, and B6083, were obtained from F. Hoffmann-La Roche Ltd. (Basel, Switzerland). Enbrel batches, 34359, 1027881, 1030760, G18414, F65452, and F40596, were obtained from Amgen Inc. (Thousand Oaks, CA, USA). Kikuzubam batches, 5445110403, 5433120509, and 5445100901, and Infinitam (drug substance) batches, ETPP12001, ETPP12002, ETPP12003, and ETPP12005 from Probiomed S.A. de C.V. (Mexico City, Mexico) were used for the study.

### 2.2. Instrumentation

A DU640 UV-Vis Spectrophotometer from Beckman Coulter Inc. (Brea, CA) and a Nanodrop 2000 UV-Vis Spectrophotometer from Thermo Fisher Scientific Inc. (Waltham, MA) were used for DTNB and protein concentration determinations. A Fluorolog 3 spectrofluorometer from Horiba Jobin Yvon (Kyoto, Japan), equipped with a Xenon arc lamp of 450 W, was used to obtain fluorescence data (steady state). All sample incubation treatments were carried on a Thermomixer Comfort from Eppendorf AG (Hamburg, Germany). Structural analyses were performed in a Circular Dichroism J-815 spectrometer from Jasco International Co. Ltd. (Tokyo, Japan). Intrinsic Trp fluorescence lifetime measured by time-correlated single photon counting (TCSPC) was performed using a 288 nm pulsed diode light source, with pulse duration <1.2 ns. Right angle emission was detected at 333 nm with 1 MHz rate using a Fluorolog 3 spectrofluorometer from Horiba Jobin Yvon. Bioassays absorbance was measured using Spectramax Plus384 microplate spectrophotometer from Molecular Devices (Sunnyvale, CA) using Soft Max Pro software.

### 2.3. Methods

#### 2.3.1. Placebos

Placebo used for Rituximab included NaCl 150 mM and 0.14 mM polysorbate 80 in citrate buffer 29 mM pH 6.5, while placebo used for Etanercept was manitol 22 mM and sucrose 3 mM in 10 mM Tris buffer pH 7.4.

#### 2.3.2. Sample Preparation

Protein samples were concentrated up to 100 mg·mL^−1^ and dialyzed against their corresponding placebos using 30 kDa centricons spin columns at 14,000 g for 15 min. Etanercept samples for the analysis under denaturing-reducing conditions were additionally digested with Neuraminidase using 20 U per mg of protein. Protein concentration was therefore adjusted to 20, 7.5 and 1 mg·mL^−1^ for native, denaturing, and denaturing-reducing conditions for DTNB method, respectively. 100 mM sodium phosphate and 5 mM EDTA buffer solution at pH 8.0 were used for DTNB method and 100 mM sodium phosphate, 150 mM sodium chloride, and 5 mM EDTA buffer solution at pH 7.0 were used for DLM method. Protein concentration for DLM method was adjusted to 1 mg·mL^−1^ either under native or denaturing conditions. For denaturing conditions of both methods PBS with GdnHCl was added to samples to give a final concentration of 5 M, while for denaturing-reducing conditions GdnHCl was taken up to 7.2 M and 10 mM DTT, and samples were incubated for 30 min at 30°C. Once samples were reduced, DTT was eliminated by buffer exchange with 100 mM sodium phosphate, 5 mM EDTA buffer solution at pH 8.0, passed over NAP-5 columns. Protein concentration under denaturing-reducing conditions was estimated at this step, prior derivatization. All solutions including placebos were degassed by sonication for 30 min and bubbled with nitrogen during 5 min prior its use, following previous reports that used argon during 15 minutes [17, 18].

Placebos were treated like samples for both methods, and absorbance and fluorescence were measured as described below.

HSA was diafiltered against purified water, using 30 kDa centricons, until triptofanate acetate was eliminated. 1 mg of purified HSA was treated with 2.5 mM DTT on PBS at pH 7.0 for 30 min at 22°C. DTT was eliminated by buffer exchange with PBS at pH 7.0, in NAP-5 columns. DTT was added in order to reduce sulfenic acids from Cys 34 to sulfhydryl groups. Three independent triplicates were derivatizated with DLM.

#### 2.3.3. Thiol Derivatization

Stock solutions of DTNB and DLM derivatization agents were diluted with DMSO. Derivatization was carried at a final concentration of 500 *μ*M of DTNB and 1 h incubation at 30°C. Absorbance was measured at 412 nm and 280 nm in 1 cm QS quartz cuvettes [11].

DLM derivatization was carried at a final concentration of 0.125 mM and 2 h incubation at 25°C. Extensive dialysis against 100 mM sodium phosphate, 150 mM sodium chloride, and 5 mM EDTA buffer solution at pH 7.0, (phosphate buffer solution (PBS)) using 10 kDa Slide-A Dialysis units, was performed after derivatization. Protein concentration under native and denaturing conditions was adjusted to 1 mg·mL^−1^ using 100 mM sodium phosphate, 150 mM sodium chloride, 5 mM EDTA buffer, and GdnHCl 5 M solution at pH 7.0. DLM fluorescence emission was measured at 518 nm, exciting at 493 nm in 3 mm QS quartz cuvettes at 25°C ± 1°C as previous studies recommended [14, 19, 20].

#### 2.3.4. Rituximab, Etanercept, and Glatiramer Acetate Amine Neutralization under Native Conditions

To neutralize amines under native conditions, 50 *μ*L of a mixture 50 : 50 (v/v) phenyl isothiocyanate, DMSO, was added into 10 aliquots of 5 *μ*L with continuous stirring to 0.4 mL of a solution at 2.5 mg/mL of protein in carbonate buffer (0.1 M pH 9.0). Samples were incubated at 5 ± 3°C for 8 h, in the dark. Afterwards, the buffer was exchanged with 15 mM sodium azide in PBS using NAP-5 columns recommended by [21]. Samples were derivatized with DLM as described before (see Section 2.3.3).

#### 2.3.5. DLM Nonspecific Interaction with Rituximab, Etanercept, and Glatiramer Acetate under Native Conditions

Prior derivatization, DLM was taken from a stock solution at 12.5 mM and was allowed to react with 50 *μ*L of 50 mM cysteine in PBS for two hours at 25°C, 600 rpm. Sample preparation and derivatization were done as described using cysteine reacted with DLM instead of DLM (Sections 2.3.2 and 2.3.3); expecting not having covalent reactions against proteins, DLM-cysteine was left to form Van der Waals or hydrophobic interactions with proteins.

#### 2.3.6. Circular Dichroism Measurements

Circular dichroism studies were carried out in a Jasco-815 spectropolarimeter, using a modified method previously reported [15]. Etanercept and Rituximab samples were diluted to 0.1 mg·mL^−1^ for far-UV CD spectra (190–300 nm) in a 0.1 cm quartz cell and to 3.3 mg·mL^−1^ for near-UV CD spectra (240–350 nm) either under native, denaturing, or denaturing-reducing conditions. Samples were diluted with 10 mM phosphate pH 7.0 for native conditions and with 10 mM phosphate and 5 M GdnHCl buffer solution pH 7.0 for denaturing conditions, while for denaturing-reducing conditions samples were treated with 10 mM DTT for 30 min at 25°C and buffer exchanged using NAP-5 columns against 10 mM phosphate buffer solution and GdnHCl 0.5 M pH 3.0. Samples were reconstituted to 10 mM phosphate buffer solution and GdnHCl 3 M pH 3.0 final concentration. Spectrum was acquired as an average of 3 scans using 0.01 (far-UV CD) and 0.1 cm (near-UV CD) quartz cuvettes at 1 nm data pitch, 1 nm bandwidth, and 50 nm·min^−1^ of scan speed. Each condition buffer was used as blank for each spectrum.

#### 2.3.7. Intrinsic Trp Fluorescence Lifetime (TCSPC)

Measurements were done in a Fluorolog 3 spectrofluorometer from Horiba Jobin Yvon (Kyoto, Japan), equipped with a Xenon arc lamp. Etanercept samples were adjusted to 2 mg·mL^−1^ with 10 mM phosphate buffer solution at pH 7.0 and measured. Data analysis for TCSPC was done using DAS6 software; fitting results were adjusted using 2 exponential decays for least squares regression. Fluorescence lifetime was obtained as amplitude weighted mean of exponential decays [16].

#### 2.3.8. CDC Bioassays with Rituximab

This bioassay was performed as Brezski and collaborators [22] with minor changes. Briefly, cell antiproliferation was induced by Rituximab through complement-dependent cytotoxicity (CDC) to CD20 expressing cells (WIL2-S, ATCC CRL-8885) in the presence of human serum complement (Quidel, CA, USA). Cells viability, following CDC treatment with Rituximab, was measured using Alamar Blue probe (Promega, WI, USA). Rituximab potency was expressed against a reference standard of 100% potency.

#### 2.3.9. Bioassays for Neutralization of TNF-*α* with Etanercept

Neutralizing activity of Etanercept over TNF-*α* was measured as the viability of A375 cell line treated with actinomycin D [23, 24]. Cells were seeded in triplicate at 5 × 10^5^/well into a 96-well plate in DMEM medium supplemented with 10% (v/v) FBS and then were incubated for 24 h, 37°C at 5% CO_2_. Medium containing TNF-*α* at 21 ng/mL was added to the cell culture (final concentration 7 ng/mL); then, Etanercept dilutions within the range of 5.2 to 60.0 ng/mL and actinomycin D at 390 ng/mL (final concentration 130 ng/mL) were added to the cell culture. Cells were incubated for additional 18 h at 37°C, 5.0% CO_2_, and then fixed with formaldehyde 5% adding 50 *μ*L to each well and dyed with 10% violet crystal. Absorbance was measured at 540 nm with a 690 nm reference filter. The ED_50_ value was calculated by four-parameter logistic curve fit using Soft-Max Pro software. Enbrel value was taken as 100% potency.

### 2.4. Method Validation (Table 1)

#### 2.4.1. System Suitability

DTNB method defined acceptance criteria were as follows. (1) Determination coefficient for L-cysteine HCl standard curve (5–200 *μ*M) must be >0.980. (2) Absorbance measurement at 412 nm from placebo (blank), water, and a thiol-free protein must be <0.005 AU. (3) Cys/Protein molar ratio under denaturing-reducing conditions of somatropine CRS standard must be 4.0 ± 0.5.

DLM method defined acceptance criteria were as follows. (1) Determination coefficient for DLM standard curve (0.1–7.5 *μ*M) must be >0.980. (2) Fluorescence measurement for placebo, water, and a thiol-free protein must be <20,000 CPS.

#### 2.4.2. Precision

*Repeatability*. Sextuplicate samples at 0.4 and 1.0 mg·mL^−1^ for Etanercept and Rituximab, respectively, were analyzed with DTNB and DyLight 488 Maleimide methods. The intermediate precision aws that two different analysts measured Etanercept and Rituximab samples in two different days. (Etanercept for DTNB method and Rituximab for DLM method) Relative standard deviation ≤10% was expected.

#### 2.4.3. Accuracy

Triplicates of somatropine CRS and HSA at 1 mg·mL^−1^ were prepared under denaturing-reducing and native conditions, respectively. HSA was treated with 2.5 mM DTT, 30 min, 22°C prior analysis, in order to reduce oxidized cysteines. Recovery percentages, expected from 90 to 110% and 60 to 140%, were calculated against theoretical Cys/Protein molar ratio of 4 and 1, for somatropine and HSA, respectively. Somatropine and HSA concentrations were determined from absorbance at 280 nm, using *ε* values of 0.82 g·L^−1^·cm^−1^, 0.531 g·L^−1^·cm^−1^ and molecular masses of 22, 124 g·mol^−1^ and 66, 470 g·mol^−1^, respectively.

#### 2.4.4. Specificity

Placebos, somatropine CRS at 1 mg·mL^−1^, GA (negative control, thiol-free peptide) at 20 mg·mL^−1^, and 10 kD protein marker at 1 mg·mL^−1^ were analyzed under denaturing-reducing and native conditions. Absorbance and fluorescence measurements according to the system suitability were expected.

#### 2.4.5. Quantification Limit

The lowest concentration level for DTNB and DLM method curves with a RSD <20% was established as limit of quantification.

#### 2.4.6. Standard Curves and Linearity

Cysteine stock solutions (1.5 mM) were done gravimetrically, diluted to 5 *μ*M, 10 *μ*M, 25 *μ*M, 50 *μ*M, 100 *μ*M, and 200 *μ*M, and analyzed in triplicates using DTNB method.

DLM was incubated with PBS, 50 mM cysteine for 2 h at 25°C. Dilutions to 7.5 *μ*M, 5 *μ*M, 3.75 *μ*M 1.85 *μ*M, 1 *μ*M, 0.25 *μ*M, and 0.1 *μ*M were analyzed in triplicates using DLM method. DLM concentration was determined by absorbance at 493 nm using an extinction coefficient of 70,000 M^−1^·cm^−1^. Determination coefficient (*r* ^2^) > 0.980 and slope ≠ 0 were expected.

### 2.5. Statistical Analysis

Two group sample comparisons were done by unpaired and two-tailed Student's *t*-test. Homogeneity of variance was tested using *F*-max test. *t*-test comparison was calculated depending on *F*-max test, using Microsoft Excel software. Differences were considered significant at *P* < 0.05. Confidence intervals were calculated at 95%. Error bars are depicted as standard error of mean (±SE).

### 2.6. Thiol/Protein Ratio Calculations

For DTNB method, thiol concentration was calculated from cysteine standard curves depending on the specific condition related to the assay. Absorbance at 412 nm was recorded and interpolated into curve made with phosphate buffer 0.1 M, pH 8.0, for native and denaturing-reducing conditions, while curve made with phosphate buffer 0.1 M and 5 M GdnHCl, pH 8.0, was used for denaturing conditions.

For DLM method, fluorescence at 518 nm was recorded and interpolated into curve. This curve was made with phosphate buffer 0.1 M and 5 M GdnHCl, pH 7.0, and was used either for native or denaturing conditions.

Cysteine and protein mass of all samples were adjusted to nanomoles per sample. Cysteine nanomoles were divided by the protein nanomoles to obtain Cys/prot molar ratio. Averages of independent triplicates were reported.

## 3. Results

### 3.1. Native Conditions

DTNB method showed no difference between placebo (blank) and glatiramer acetate (GA) (thiol free peptide) at 20 mg·mL^−1^, with absorbance values of 0.0005 and 0.0014 AU for placebos and GA, respectively. Glatiramer acetate (GA), a random copolymer of tyrosine, lysine, alanine, and glutamic acid, was used as negative control. Fluorescence measurements about 1600 and 5000 cps were obtained for placebo and 10 kDa protein marker, respectively.

Linear range using L-cysteine as standard was demonstrated from 5 *μ*M to 200 *μ*M derivatizing with DTNB, while for DLM method, using DyLight 488 Maleimide linked to L-cysteine as standard, it was demonstrated from 0.1 *μ*M to 7.5 *μ*M (Table 1).

#### 3.1.1. Rituximab

Analysis of three different batches of Mabthera and Kikuzubam shows no statistical differences for their Cys/Protein molar ratios, with *P* values of 0.6233 and 0.7499 for DTNB and DLM methods, respectively. Cys/Rituximab average molar ratios were 0.018 and 0.020 for Mabthera and Kikuzubam, respectively, using DTNB method with a confidence interval at 95% from 0.0253 to 0.0096 for Mabthera, while 0.064 and 0.068 average molar ratios were obtained for the products using DLM method with a confidence interval at 95% from 0.0865 to 0.0408 for Mabthera (Tables 2 and 3).

#### 3.1.2. Etanercept

Enbrel and Infinitam showed no statistical difference with DTNB method (*P* = 0.0758), while DLM method reveals statistical differences among products (*P* < 0.001). Cys/Etanercept average molar ratios were 0.024 and 0.014 for Enbrel and Infinitam, respectively, using DTNB method with a confidence interval at 95% from 0.0361 to 0.0120 for Enbrel, while 0.084 and 0.047 average molar ratios were obtained for the products using DLM method with a confidence interval at 95% from 0.0978 to 0.0710 for Enbrel (Tables 2 and 3).

### 3.2. Denaturing Conditions

Like native conditions, GA was used as negative control, with its absorbance values lower than 0.002 AU, for DTNB method, while fluorescence from 10 kDa protein marker was below 5000 cps for DLM method (Table 4).

Linear range, using L-cysteine as standard, was demonstrated from 5 *μ*M to 100 *μ*M for DTNB method and, using DLM linked to L-cysteine as standard, was from 0.1 *μ*M to 7.5 *μ*M for DLM method (Table 1).

#### 3.2.1. Rituximab

Statistical analysis shows equality between Mabthera and Kikuzubam with *P* values of 0.4022 and 0.8027 for DTNB and DLM methods, respectively. Cys/Rituximab average molar ratios of 0.602 and 0.558 with a confidence interval of 95% from 0.6977 to 0.5068 for Mabthera were obtained using DTNB method, while 1.149 and 1.076 average molar ratios were obtained for Mabthera and Kikuzubam, respectively, using DLM method with a confidence interval from 1.7010 to 0.5973 for Mabthera (Tables 2 and 3).

#### 3.2.2. Etanercept

Statistical equality was obtained for Enbrel and Infinitam with *P* values of 0.2450 and 0.7983 using DTNB and DLM methods, respectively. Cys/Etanercept mean molar ratios of 0.447 and 0.498 for Enbrel and Infinitam, respectively, with a confidence interval at 95% from 0.5388 to 0.3548 for Enbrel, were obtained with DTNB method. Mean molar ratios of 0.7787 and 0.7618 using DLM method were obtained for Enbrel and Infinitam, respectively, with a confidence interval at 95% from 0.9488 to 0.6087 for Enbrel (Tables 2 and 3).

### 3.3. Denaturing-Reducing Conditions

Unlike native or denaturing conditions, the analysis of free thiols under denaturing-reducing conditions is a direct measure of the total cysteines residues within the protein. Somatropine CRS was used as positive control to verify the measurement, with an expected Cys/Somatropine mean molar ratio of 4 (Table 4).

#### 3.3.1. Rituximab

The expected number of cysteine residues in Rituximab, as an IgG isotype I, is 32. DTNB method results for Mabthera and Kikuzubam were 31.6 ± 0.8 and 30.8 ± 1.5, respectively. Statistical analysis showed a *P* value of 0.1475 between products with a confidence interval at 95% from 32.3 to 31.0 for Mabthera (Table 2).

#### 3.3.2. Etanercept

Theoretical content of cysteine residues in Etanercept according to the primary sequence is 58. DTNB method results for Enbrel and Infinitam under denaturing-reducing conditions were 56.6 ± 2.5 and 58.9 ± 1.4, respectively. Statistical analysis reveals a *P* value of 0.0229 with a confidence interval at 95% from 58.6 to 56.6 for Enbrel (Table 2).

### 3.4. Method Validation

Characteristics were chosen for method validation, according to ICH Q2 R1 guideline [25]. DTNB method, complied with all the previously designed acceptance criteria, was based on results obtained during analytical development. Validation results are summarized in Table 1.

Repeatability results show better performance for DLM method than for DTNB method under native conditions; RSD was decreased from 17.3% to 13.3%. Also 20 times less protein concentration was used (1 mg·mL^−1^ instead of 20 mg·mL^−1^) and a lower quantification limit (5 *μ*M to 0.1 *μ*M) was observed (Table 1).

Linearity was evaluated using the solution buffers according to the different conditions (native and denaturing; for denaturing-reducing conditions samples were previously buffer exchanged with native conditions' buffer) as components can affect standard curve slopes. Calibration curve slopes for cysteine standard using DTNB method under denaturing conditions and native conditions showed a difference of 27.3% (Table 1).

Calculated recovery percentage under denaturing-reducing conditions, using Somatropine CRS, was 99.5% for DTNB method. Calculated recovery percentage under native conditions, using HSA, for DLM method was 101.0% (Table 4).

## 4. Discussion 

Analysis of free thiol groups under native conditions of the evaluated Rituximab products (Kikuzubam and Mabthera) showed equivalent Cys/Protein molar ratios, for DLM (*P* = 0.7499) and DTNB method (*P* = 0.6233), suggesting that both molecules have comparable structures and physicochemical integrity [26] (Tables 2 and 3).

Cys/Protein molar ratios within the range of 0.010 to 0.020 have been reported for therapeutic mAb's under native conditions using DTNB as derivatizing agent [26]. These values, derived from current commercial products, predicted to maintain biological, physicochemical, and structural integrity, can be used as a reference range, where the preservation of the folded structure inhibits the exposure of sulfhydryl groups and their oxidation to sulfenic, sulfinic, or sulfonic groups while restraining disulfide bridges cleavage. Cys/Protein molar ratios of Rituximab products, using DTNB method, rely within the aforementioned range, with 0.020 and 0.018 for Kikuzubam and Mabthera, respectively (Table 2). However, DLM method revealed higher Cys/Protein molar ratios, although comparability between products was confirmed, with 0.068 and 0.064 for Kikuzubam and Mabthera, respectively (Table 3). The enhancement of fluorescence was expected from DLM noncovalent interactions towards hydrophobic patches within the protein or by the covalent reaction against amine substituents [27], although DLM reaction against primary amines was diminished using a low DLM concentration [27] and a buffer at pH <7.5 [20]. Nevertheless, DLM method results for glatiramer acetate (thiol-free peptide), used as a negative control, revealed that fluorescence response was only reduced at placebo-response levels after phenylisothiocyanate titration against its amine groups (Table 5). It was possible to make this phenomenon evident because of the basic nature and high lysine concentration of glatiramer acetate (pI around 10.5), showing a Cys/Protein molar ratio of 0.045 under native conditions, far from the 0.003 ratio after amine neutralization, where DLM-amine interactions were avoided and expected null response was obtained (Table 5). Nonspecific interactions of DLM were tested using cysteine to neutralize DLM prior contact with protein, resulting in a Cys/Protein molar ratio <0.001 for glatiramer acetate, under native conditions, which is an indicative of absence of those interactions (Table 5). Instead, Cys/Protein molar ratios from Rituximab and Etanercept tested with cysteine neutralized DLM were 0.023 and 0.035, respectively, enlightening a protein-specific DLM interaction, related to three-dimensional conformation, polarity, and electrical charge. Overall, this explains the high Cys/Protein molar ratios observed for DLM method and the unsuitability of arithmetical corrections. It is worth to mention that Maleimide artifacts are major drawbacks of DLM method, although the fundamental reasons need to be understood and overcome, which is out of the scope of this paper.

Under denaturing conditions, Cys/Protein molar ratios of both Rituximab products show no statistical difference, using DLM (*P* = 0.8027) and DTNB methods (*P* = 0.4022). This confirmed structural similarity among products because of the content of buried thiol substituents, coming from nonbonded or broken disulfide bridges [28], revealing the same susceptibility towards degradation (Tables 2 and 3). Although, under denaturing conditions, Cys/Protein molar ratios are 15 to 30 times higher than native conditions (Tables 2 and 3), no impact in the biological activity of Rituximab products was detected (Table 6). This is in accordance with published data that shows no impact on the biological activity of a mAb that binds CD20 containing unpaired cysteines [28].

Once fully denatured and reduced, 32 exposed cysteine residues are expected in Rituximab according to its primary sequence. Cys/Protein molar ratio measurements, under denaturing-reducing conditions using DTNB method, showed that Mabthera and Kikuzubam are comparable (*P* = 0.1475) and have a mean value of 31.6 and 30.8 cysteine residues, respectively (Table 2). Kikuzubam difference against the theoretical value (3.8%) relies within the RSD of the method (Table 1).

**Figure 1:**
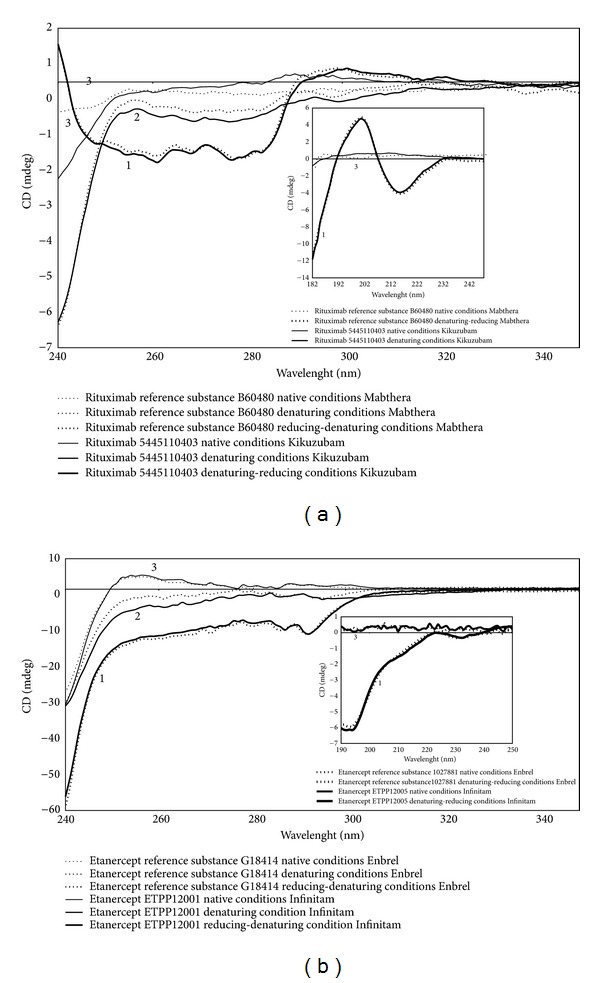
Far- and near-UV CD spectra of (a) Rituximab and (b) Etanercept products under (1) native, (2) denaturing, and (3) denaturing-reducing conditions. Dotted lines represent Mabthera (Rituximab) and Enbrel (Etanercept), while continuous lines represent Kikuzubam (Rituximab) and Infinitam (Etanercept) representative batches. Main plot shows near-UV CD spectra (240–350 nm) and graphic insert shows far-UV CD spectra (190–300 nm). Near- and Far-UV characteristic spectra of Rituximab are shown in (a) and insert shows antiparallel *β*-sheets coming from CH_1_, CH_2_, and CH_3_ and V_L_ and V_H_ domains. Etanercept spectra (b) show an irregular structure directed probably by complex and diverse glycan substituents. Each sample was run in triplicate; average is represented as single spectrum (details are described in Section 2.3.6).

Quantitation of buried thiol substituents that come from broken and unpaired disulfide bridges or the total cysteine content within denatured and denatured-reduced Rituximab products, respectively, was suggested from structural analysis by CD spectra. As seen from Figure 1 Rituximab secondary structure (far CD-UV spectra), that comprises mainly domains of two beta sheets linked by a disulfide bridge and compressed by *β*-antiparallel barrel [29], was lost when treated with both denaturing and reducing agents. This was also observed for tertiary structure response (near CD-UV spectra), as signal from native condition was diminished under denaturing conditions and lost when Rituximab was treated with a reducing agent, thus confirming structural similarity among Rituximab products.

CD analyses for Etanercept also confirmed structural similarity among the evaluated products, Infinitam and Enbrel, revealing comparable loss of structure from native to denaturing-reducing conditions for near CD-UV and far CD-UV spectra (Figure 1). Thiol analysis under native conditions, using DLM method, showed a higher content of free and exposed thiols for Enbrel than Infinitam, while DTNB method showed no statistical difference between Cys/Protein molar ratios of Etanercept products (*P* = 0.0758) (Tables 2 and 3). Statistical analysis of Cys/Protein molar ratios for DLM method revealed a *P* value <0.001, suggesting a higher amount of denatured molecules in Enbrel batches or different glycosylation patterns that could affect thiol determination (steric hindrance). However intrinsic Trp fluorescence lifetime (Table 7) and biological activity through TNF-*α* neutralization assays (Table 6) revealed no significant differences among products. These results make evident the same DLM artifacts seen for Rituximab and glatiramer acetate, mostly relevant for the measure of Cys/Protein molar ratios <1.0. Actually, under denaturing conditions, statistical equality was observed using DLM (*P* = 0.7984) and DTNB (*P* = 0.2450) methods.

For the analysis of Etanercept products, under reducing conditions, samples were previously desialylated with neuraminidase in order to avoid the electrostatic repulsion of DTNB dye against sialic acids and to diminish steric hindrance effects that could came from the high glycan density of Etanercept (up to 30% of the total molecular mass) and its high negative charge density (0.1 to 0.2 nmoles of sialic acids per mole of Etanercept). Neuraminidase treatment improved the measurement from the initially obtained Cys/Protein mean molar ratio around 20, far from the expected value of 58, to mean molar ratios of 56.6 and 58.9 for Enbrel and Infinitam, respectively. Enbrel difference against the expected value was 2.5%; thus it is meaningless to establish a difference among products, despite the fact that both groups are not statistically equal (*P* = 0.0229). These results show that both products are comparable based on their equal behavior. Thus differences against theoretical values of Rituximab and Etanercept products are due to sample properties, possibly due to their vulnerability to be oxidized, although degassing of dissolved oxygen for all buffers solutions was performed. However, the observed difference in both pharmaceutical ingredients is lower than the RSD of the method (Tables 2 and 3).

Validation of DLM and DTNB methods complies with the specified acceptance criteria (Table 1). Specificity test for DTNB method using glatiramer acetate (negative control) showed a mean absorbance of 0.0014, around 10 times below than Etanercept under native conditions, thus being acceptable and confirmatory of all results (Table 4). For DLM method the use of glatiramer acetate was not possible because of nonspecific reactions. 10 kDa protein marker was proved to be suitable as negative control, but absolute Cys/Protein mean molar ratios need to be taken carefully when being <1.0; although being repeatable (RSD 17.3%) and consistent, still they are beneath the artifacts values. It is worth to mention that high RSD for DTNB and DLM, in Tables 2 and 3, comes mainly from batch to batch variability than method uncertainty; therefore they can be useful for measured dispersion between batches and also in new molecules process development.

Accuracy for DTNB method under denaturing-reducing conditions was assured using Somatropine CRS as positive control; results showed a Cys/Protein mean molar ratio of 3.98, which corresponds to the theoretical value of 4 (99.5% recovery) (Table 4). Measurements with DLM method, using HSA as positive control, proved to be accurate around the theoretical value of 1 (101.0% recovery), which is above nonspecific DLM-protein values (Tables 4 and 5). Validation results are summarized in Table 1.

Therefore, orthogonal DLM and DTNB methodologies showed that structural integrity of biopharmaceuticals produced by different manufacturers is comparable, Kikuzubam versus Mabthera and Infinitam versus Enbrel, respectively. Spectroscopic methods confirmed these results.

During method development several drawbacks were identified and some overcame; these arise mainly from the lack of detailed descriptions in the published procedures; Issues, causes, and solutions are listed in a troubleshooting guide (Table 8) that highlights aspects of the methodology here described.

## 5. Conclusions

DTNB and DLM methods were proven to be suitable according to validation results for the characterization and comparability analyses of free thiol groups in biopharmaceuticals, whereas DLM method showed to be at least 50 times more sensitive than DTNB method (demonstrated by comparison of curves, quantitation limit). However, it could not be used in the low range thiol quantification because several interactions of DLM towards protein amines and hydrophobic patches are present.

Using both techniques, comparability between Rituximab and Etanercept products coming from different manufacturers was proved for thiol analysis under native, denaturing, and denaturing-reducing conditions and confirmed by CD, TCSPC, and biological activity assays. Each selected condition was chosen as a measure of the protein either: intact, denatured without disulfide bridges disruption, or denatured reduced with all its thiol substituents exposed.

Nowadays, to the best of our knowledge, several studies report the use of DTNB and DLM methods for different purposes; however in this study we recommended some suggestions to reduce variability of measurements, high background, over thiol quantification and also increase the method sensitivity in order to have successful method performance. Previous information has been used to overcome drawbacks. Here we reported the capabilities of both colorimetric and fluorometric methods to determine free reactive thiols under different protein conditions, in order to demonstrate the protein structural correspondence between biosimilars and innovator drug products.

## Figures and Tables

**Table 1:** Validation results for DTNB and DLM methods.

Validation test	Acceptance criteria	Method result	
		DTNB	DyLight 488
Precision (repeatability)	<20%	RSD: 2.52% (denaturing-reducing conditions). RSD: 17.3% (native conditions).	RSD: NT% (denaturing-reducing conditions). RSD: 13.3% (native conditions).

Accuracy	Total recovery between 95 and 105% in respect to theoretical value.	99.5% recovery using Somatropin (Cys/Protein ratio 3.98)	101.0% recovery using HSA (Cys/Protein ratio 1.01)

Intermediate precision	RSD < 10%	RSD: 3.8%	RSD: 0.66%

Linearity	*r* ^2^ ≥ 0.980 Ordinate = 0	*r* ^2^ = 0.999 native and denaturing-reducing conditions. Slope = 0.01285 *μ*M^−1^ *r* ^2^ = 0.983 denaturing conditions. Slope = 0.0093 *μ*M^−1^ Forced through zero.	*r* ^2^ = 0.990 native and denaturing conditions. Slope = 465,834 *μ*M^−1^ Forced through zero.

Quantification limit	Report value	5 *μ*M	0.1 *μ*M

Selectivity	Cysteine-free protein has the same signal of placebo.	GA: 0.0010 Placebo: 0.0005	PM: 0.0005 Placebo: 0.0005

NT%: not tested.

**Table 2:** Thiol quantification using DTNB method. Rituximab and Etanercept free thiols under native, denaturing, and denaturing-reducing conditions using DTNB method. Uncertainty values are presented in SD (*n* = 9).

	Condition	Mabthera	RSD	Kikuzubam	RSD	*P*
Rituximab	Native	0.0175 ± 0.010	55.0	0.0200 ± 0.012	58.4	0.6233
	Denaturing	0.602 ± 0.117	19.4	0.558 ± 0.102	18.4	0.4022
	Denaturing/reducing	31.6 ± 0.8	2.7	30.8 ± 1.5	4.7	0.1475

	Condition	Enbrel	RSD	Infinitam	RSD	*P*

Etanercept	Native	0.0241 ± 0.015	61.5	0.0137 ± 0.005	37.6	0.0758
	Denaturing	0.447 ± 0.113	25.3	0.498 ± 0.060	12.1	0.2450
	Denaturing/reducing	56.6 ± 2.5	4.4	58.9 ± 1.4	2.4	0.0229

**Table 3:** Thiol quantification using DLM method. Rituximab and Etanercept free thiols under native and denaturing conditions using DLM method. Dispersion values are presented in SD (*n* = 9).

Protein	Condition	Mabthera	RSD	Kikuzubam	RSD	*P*
Rituximab	Native	0.0636 ± 0.028	44.1	0.0683 ± 0.033	47.8	0.7499
	Denaturing	1.149 ± 0.480	41.8	1.076 ± 0.508	47.2	0.8027

	Condition	Enbrel	RSD	Infinitam	RSD	*P*

Etanercept	Native	0.0844 ± 0.017	19.5	0.0477 ± 0.020	41.7	<0.001
	Denaturing	1.091 ± 0.207	19.0	1.067 ± 0.068	6.4	0.7984

**Table 4:** Specificity and accuracy of thiol analysis. Controls are specified for DTNB method and DLM method.

Protein	Condition	Cys/Protein molar ratio	
		DTNB	DLM
Somatropin	Denaturing/reducing	3.98 ± 0.31	NT

HSA	Native	NT	1.01 ± 0.17
Glatiramer acetate		0.001 ± 0.007	0.045^a^
Placebo Rituximab		0.0005 ± 0.001	0.0004^a^
Placebo Etanercept		0.0005 ± 0.001	0.0024^a^
Protein marker		NT	0.0005^a^

NT: not tested.

^a^SD value is less than 1 × 10^−5^.

**Table 5:** DyLight Maleimide method and nonspecific interactions tests under native conditions.

Protein	1	2	3	4
	Thiol quantification by DLM method	Nonspecific interaction (DLM neutralization)	Protein amine neutralization (protein isothiocyanate treatment)	Thiol quantification by DTNB Method
A Rituximab	0.0632 ± 0.0274	0.0230 ± 0.0001	0.0426 ± 0.0094	0.0180 ± 0.0032
B Etanercept	0.0965 ± 0.0028	0.0349 ± 0.0000	0.0229 ± 0.0000	0.0240 ± 0.0049
C Glatiramer acetate	0.0450 ± 0.0000	0.0001 ± 0.0001	0.0026 ± 0.0000	0.0010 ± 0.0024

**Table 6:** Biological potency for Etanercept and Rituximab products.

Product	Batch number	Biological potency (%)
^ a^Infinitam	ETPP12001	106
	ETPP12003	101
	ETPP12005	110

^ b^Kikuzubam	5445110403	106
	5433120509	96
	5445100901	115

^a^Biological potency measured by TNF-alfa neutralization relative to Enbrel.

^b^Biological potency measured by CDC relative to Mabthera.

**Table 7:** Intrinsic Trp fluorescence lifetime measure using TCSPC for Etanercept products. Dispersion values are presented in SD (*n* = 9).

Product	Seconds (s)	*P*
Enbrel	1.30*E* − 09 ± 6.24*E* − 12	0.2784
Infinitam	1.29*E* − 09 ± 8.98*E* − 12	

**Table 8:** Troubleshooting for DTNB and DyLight 488 Maleimide methods.

Problem	Cause	Solution
Native conditions		
High variability on measurements.	Low protein quantity.	Increase protein concentration to 20–30 mg·mL^−1^.
	Samples homogeneity.	Always vortex protein solutions.
High background.	Particles in buffer solution.	Filter buffer solutions through 0.2 *μ*m hydrophilic membrane.
High sulfhydryl measurement.	Sample stress.	Avoid high temperatures and analyze samples within 12 hours once prepared. Dialyze samples at 4°C.
	Sample excipients interference.	Dialyze samples against water.

Denaturing conditions		
Low sulfhydryl measurement	pH lower than 6.5.	Verify buffer solution pH after GdnHCl addition.
High sulfhydryl measurement using DyLight 488 Maleimide.	pH higher than 7.5.	Verify buffer solution pH after GdnHCl addition.
	Overincubation.	2 hours of incubation must be enough for derivatization.
	Incomplete dialysis.	Increase dialysis time and buffer exchange.

Denaturing-reducing conditions		
Low sulfhydryl measurement.	Oxidized thiols.	Degasify all buffer solutions by sonication during 30 min and nitrogen bubbling during 2–15 min prior its use.
High background at 280 nm using DTNB.	TNB interference.	Estimate protein concentration prior derivatization.
